# A Case of Concomitant Pemphigus Foliaceus and Oral Pemphigus Vulgaris

**DOI:** 10.1007/s12105-017-0884-0

**Published:** 2018-01-16

**Authors:** Alexandra C. Perks, Paula M. Farthing, Ruth Murphy, Anne M. Hegarty

**Affiliations:** 1 Oral Medicine Unit, Charles Clifford Dental Hospital, 76 Wellesley Road, Sheffield, S10 2SZ UK; 2 Unit of Oral and Maxillofacial Pathology, The School of Clinical Dentistry, University of Sheffield, 19 Claremont Crescent, Sheffield, S10 2TA UK; 3 Department of Dermatology, Royal Hallamshire Hospital, Glossop Road, Sheffield, S10 2JF UK

**Keywords:** Pemphigus, Desmogleins, Epitopes, Autoimmune disease

## Abstract

Pemphigus is a chronic autoimmune condition that can affect multiple areas of the body. The two main subtypes of pemphigus are pemphigus vulgaris (PV) and pemphigus foliaceus (PF) which can rarely occur concurrently or even transition from one to the other. The process of transition may be explained by qualitative changes in desmoglein autoantibody profile. We present a rare case of concomitant PF and oral PV and explore the literature on transitions between pemphigus subtypes and whether this case could represent a transition from PF to PV. Furthermore, the realities of multidisciplinary patient management are discussed.

## Introduction

Pemphigus is a rare autoimmune mucocutaneous blistering condition with four variants; pemphigus vulgaris, pemphigus foliaceus, IgA pemphigus and paraneoplastic pemphigus. These all differ in their histological features and target antigens [1]. The two major types are pemphigus vulgaris (PV) and pemphigus foliaceus (PF) [2]. Histologically both are characterised by suprabasal acantholysis, however in PV this occurs in the lower third of the epithelium/epidermis whereas in PF it occurs in the upper third of the epidermis [2, 3]. This acantholysis is as a result of circulating autoantibodies targeting desmosomal cadherins within desmosomes that bind epithelial cells together [2, 4].

**Fig. 1:**
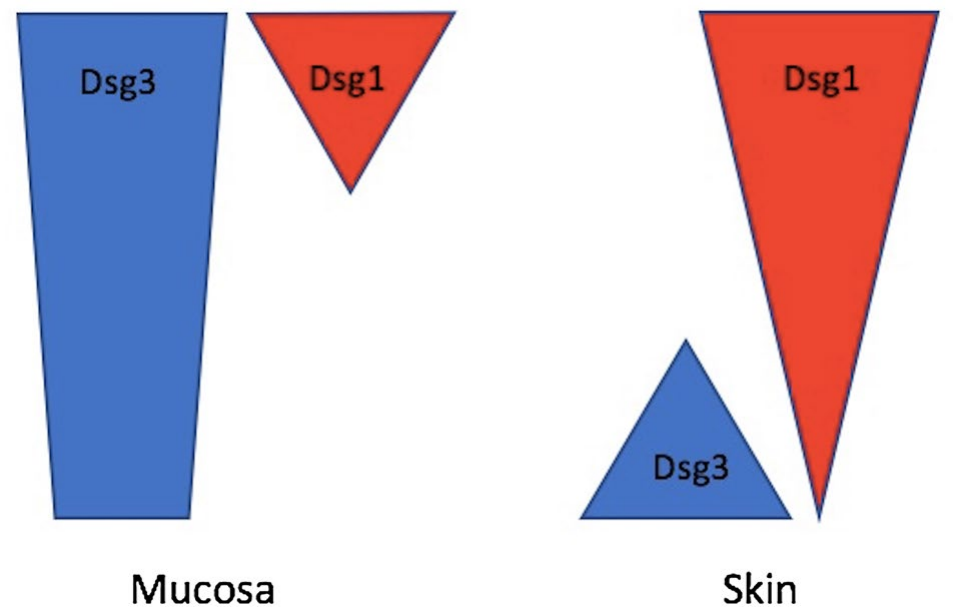
Distribution of Dsg1 and Dsg3 in mucosa and skin. In mucosa, Dsg3 is expressed highly throughout the entire epithelium, whereas Dsg1 is expressed in much lower amounts and mainly in the superficial layers. Conversely, in skin, there is only a low amount of Dsg3 which is expressed in the basal and parabasal layers only, whereas Dsg1 is expressed throughout the entire epidermis and particularly highly in the superficial layers [5, 6]

PV targets desmosomal cadherins named desmoglein 3 (Dsg3) and desmoglein 1 (Dsg1) whereas PF targets Dsg1 only. Dsg3 and Dsg1 display different expression patterns within mucosa and skin which influences the distribution of blistering [5, 6] (Fig. 1). In mucosa, Dsg3 is expressed highly throughout the entire epithelium, whereas Dsg1 is expressed in much lower amounts and mainly in the superficial layers. Conversely, in skin, there is only a low amount of Dsg3 which is expressed in the basal and parabasal layers only, whereas Dsg1 is expressed throughout the entire epidermis and particularly highly in the superficial layers [5–7].

The method by which these differing expression patterns affects the distribution of blisters is explained by the ‘Dsg compensation theory’ [5–8]. PF has only anti-Dsg1 autoantibodies, therefore in the mucosa the presence of Dsg3 can compensate for the loss of Dsg1 and no blisters are formed. In the skin, the presence of Dsg3 in the lower third of the epidermis can similarly compensate, however there is no Dsg3 in the upper third of the epidermis hence there is blister formation here [5–8]. Therefore, in PF, the oral mucosa is unaffected and blistering commonly affects the skin of the trunk, scalp and face [3, 4, 9, 10]. In the later stages of PV, both anti-Dsg1 and anti-Dsg3 autoantibodies can be displayed, and as a result no compensation can occur resulting in blisters affecting both mucosa and skin [5–8]. Therefore, in PV, blistering can affect the oral cavity, skin, pharynx, larynx, conjunctiva, oesophagus and anogenital region [4, 9, 10].

A literature search using *Pubmed, Ovid* and *Web of Science* revealed a very limited number of cases of concomitant PF and oral PV. We also reviewed the literature regarding transitions between pemphigus subtypes. Therefore, the aim of this case report is to demonstrate the management of a rare case of PF and oral PV and discuss whether it represents a rare transition from PF to PV.

## Case Report

**Fig. 2:**
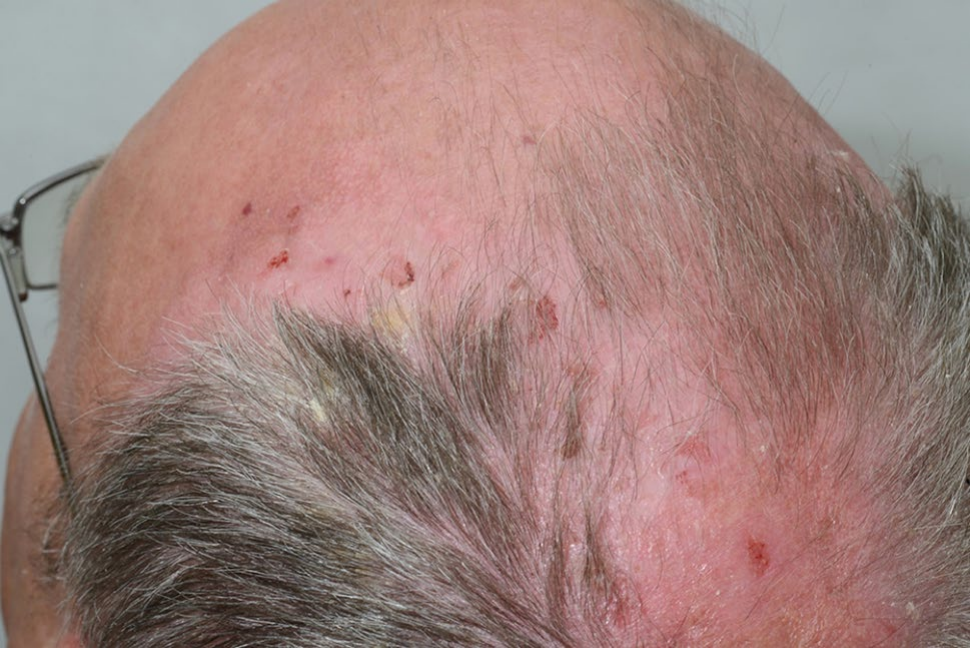
Crusted lesions on the scalp with histological diagnosis of PF

**Fig. 3:**
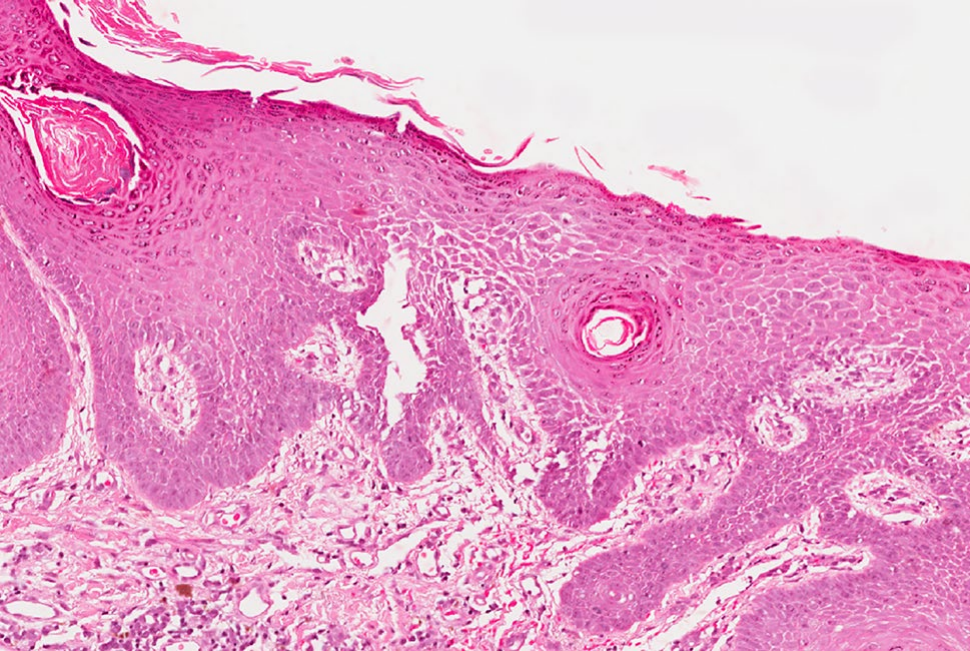
Histological image of skin from the scalp showing acantholysis in the upper third of the epidermis in PF (×20 magnification Haematoxylin & Eosin)

A 53-year-old male was referred to the Oral Medicine Department by Dermatology in July 2008, with an 8-month history of blistering and soreness of the oral mucosa. On examination, there were small erosions and ulcers present on the soft palate and bilaterally on the buccal mucosae. The patient had been under the care of Dermatology since 2003 for an itchy rash on the legs, scalp and chest. A biopsy from a crusted lesion on the scalp (Fig. 2) in 2003 revealed acantholysis of the keratinocytes in the upper part of the prickle cell layer (Fig. 3), and direct immunofluorescence studies showed IgG positivity around the upper epidermal cells. These features were consistent with a diagnosis of PF. He was started on Mycophenolate Mofetil (MMF) 500 mg twice a day and Prednisolone 10 mg once a day by Dermatology in 2006, which had resulted in excellent control of his cutaneous lesions. Prior to this, Azathioprine alone had been introduced on two occasions by Dermatology, however the patient failed to tolerate it due to nausea and vomiting. Oral involvement only began in January 2008 which required hospital admission for a short period.

His only other relevant medical history was hypertension, for which he took Nifedipine. At initial presentation, he was also taking Ranitidine and Alendronic Acid for protection against osteoporosis with prolonged systemic corticosteroid use. He was a non-smoker and had low alcohol intake.

At initial appointment, MMF dose was increased to 1 g in the morning and 500 mg in the evening for 2 weeks, and thereafter 1 g twice a day. Full blood count, urea and electrolytes and liver function tests were normal, and regular blood monitoring was carried out appropriately. In light of good response to systemic therapy and as only very small erosions/ulcers were present at this point, oral biopsy was not arranged. He was seen regularly on the joint Oral Medicine/Dermatology clinic and complete resolution of the oral lesions had occurred 5 months later. MMF 1 g twice a day was continued, however the Prednisolone dose was gradually reduced before being stopped. After ceasing Prednisolone, the patient developed an itchy dry patch of skin on the forefinger of his right hand. This was assessed by a consultant Dermatologist who clinically diagnosed Lichen Simplex Chronicus and advised him to use Elocon (Mometasone) cream.

**Fig. 4:**
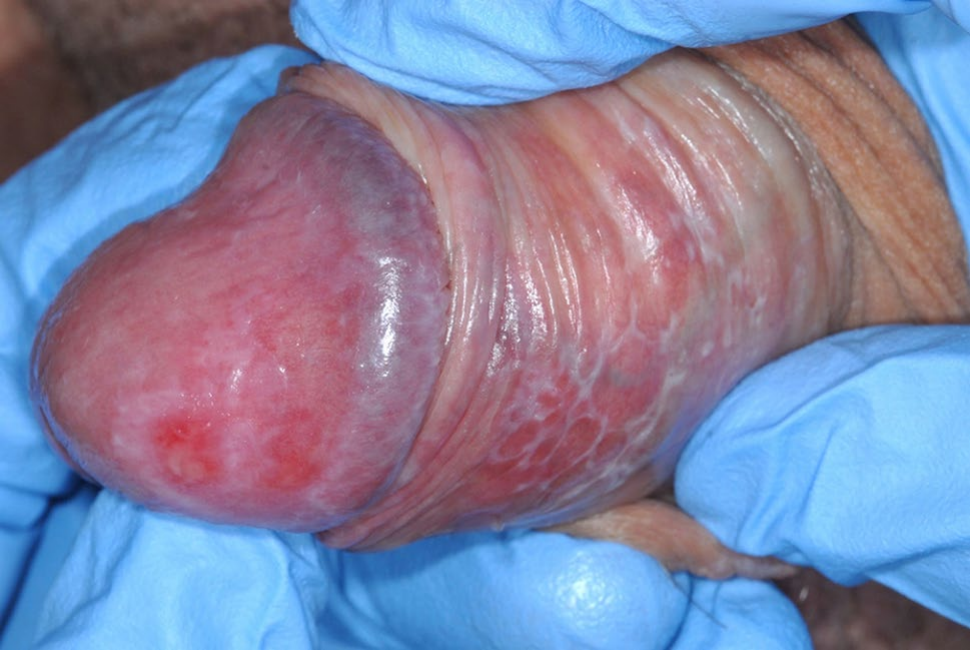
Wickham striae and erosions on the glans penis, characteristic of erosive LP

**Fig. 5:**
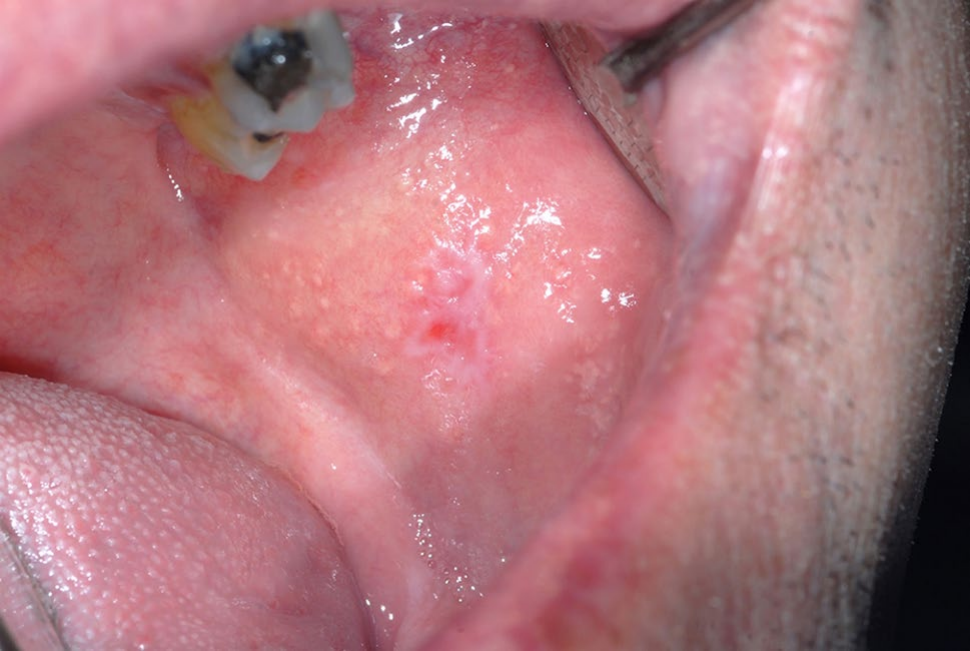
Erosion on the buccal mucosa with histological diagnosis of PV

**Fig. 6:**
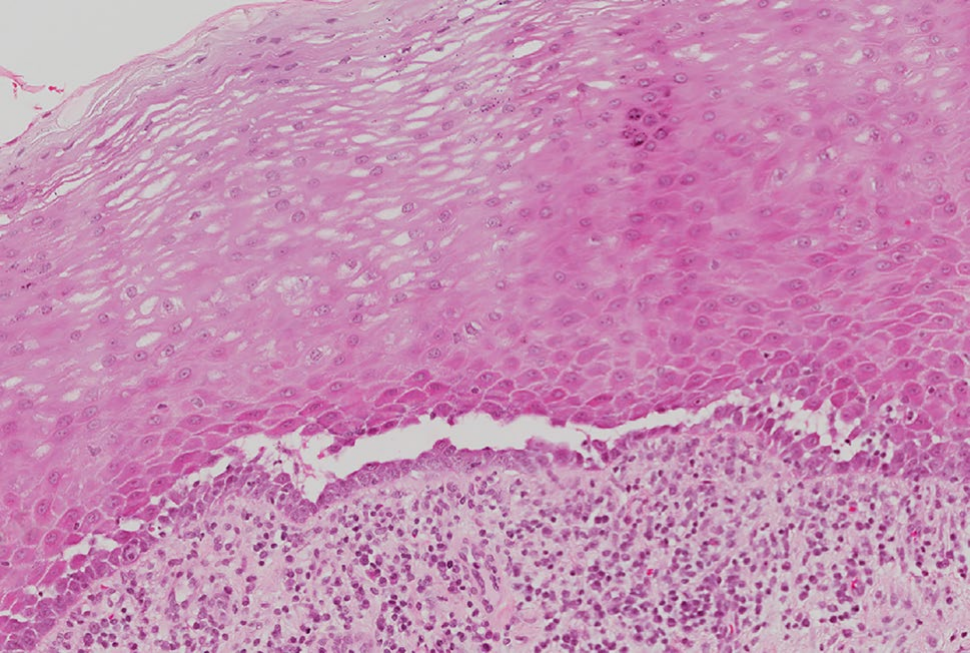
Histological image of buccal mucosa showing acantholysis in the lower third of the epithelium in PV (×20 magnification Haematoxylin & Eosin)

In 2010, the patient reported a flare in cutaneous symptoms, including scalp and genital discomfort. On examination, there were crusted lesions on the scalp. Wickham striae and erosions were seen on the glans penis (Fig. 4), therefore genital erosive lichen planus was clinically diagnosed by a consultant Dermatologist. There were also skin lesions on the arms and legs clinically resembling lichen planus. Oral lesions, more significant than seen previously, were also noted at this appointment. These consisted of an ulcer on the soft palate and an erosion in the left buccal mucosa (Fig. 5). MMF dose was increased and incisional biopsies of the buccal mucosa for histopathology and direct immunofluorescence were organised. This revealed intra-epithelial separation between prickle and basal cell layers (Fig. 6), and positive staining for IgG in the lower third of the epithelium. The features were consistent with a diagnosis of PV.

The patient proceeded to have well controlled oral PV, however problematic PF of the scalp which was managed with Xamiol gel (calcipotriol and betamethasone diproprionate) and Etrivex shampoo (clobetasol proprionate). The dose of MMF has varied depending on symptoms and currently the skin, oral mucosa and genitals are stable on MMF 1 g in the morning and 500 mg in the evening.

## Discussion

A literature search using *Pubmed, Ovid* and *Web of Science* using the search terms ‘pemphigus vulgaris’ and ‘pemphigus foliaceus’ and ‘coexisting’ or ‘coexistence’, as well as a second search using the search terms ‘pemphigus’ and ‘vulgaris’ and ‘foliaceus’ revealed a limited number of cases of concomitant PV and PF. A total of 8 papers were identified (with a total of 17 patients), however only 3 of these papers (5 patients) showed patients with both cutaneous and oral mucosal involvement [2, 11, 12]. The remaining papers involved patients with cutaneous lesions only [13–16], with one describing a case of concurrent PV and PF of the nose [17]. Of the three papers describing both cutaneous and oral mucosal involvement, only Komai et al. [2] demonstrated a histological diagnosis of both PV and PF in three patients. The other two papers only had a histological diagnosis of PV, with the diagnosis of PF being made clinically [11, 12]. This makes our case particularly novel.

**Fig. 7:**
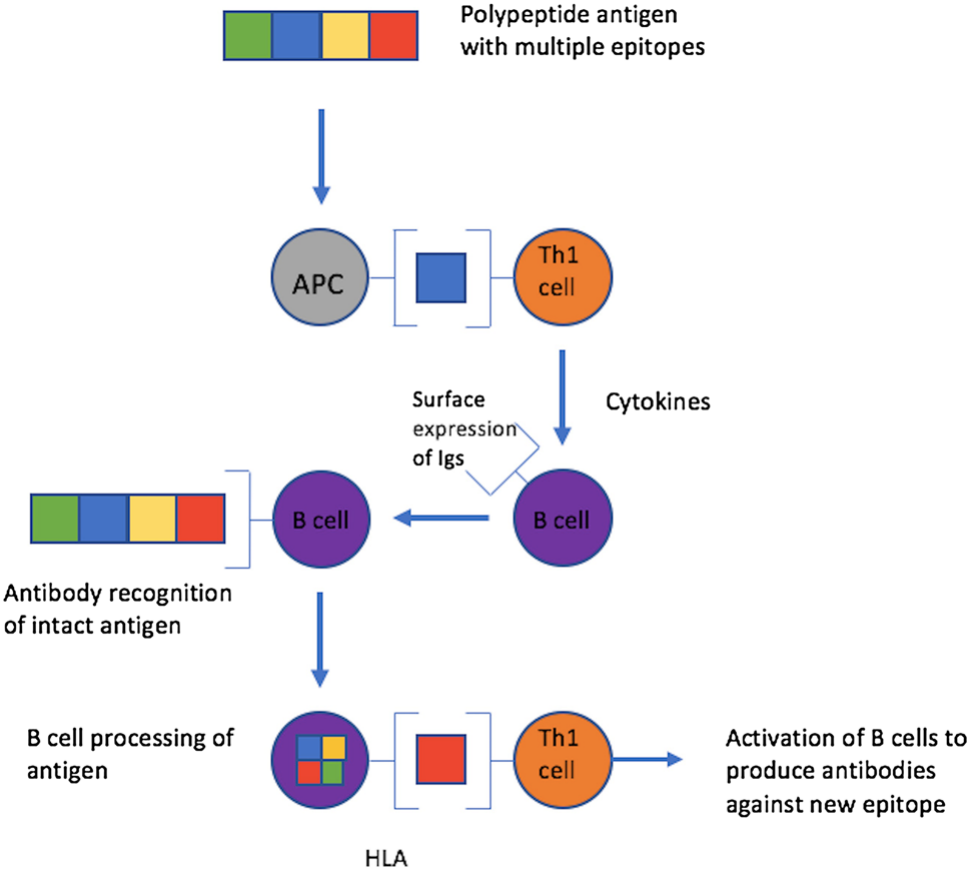
Diagram of intramolecular epitope spreading. An antigen has multiple epitopes (different coloured boxes). The antigen is processed by an antigen presenting cell (APC) and one fragment (blue square) is presented to a T-helper cell (Th1 cell). The Th1 cell releases cytokines which stimulates a B cell to produce antibodies and express antigen specific immunoglobulins on the cell surface. Surface immunoglobulin then recognises the intact antigen and it is processed by the B cell acting as an APC. A new epitope (red square) is then presented to a Th1 cell with a different antigen specificity. This initiates the production of different antibodies to a new epitope of the same antigen [23]

**Fig. 8:**
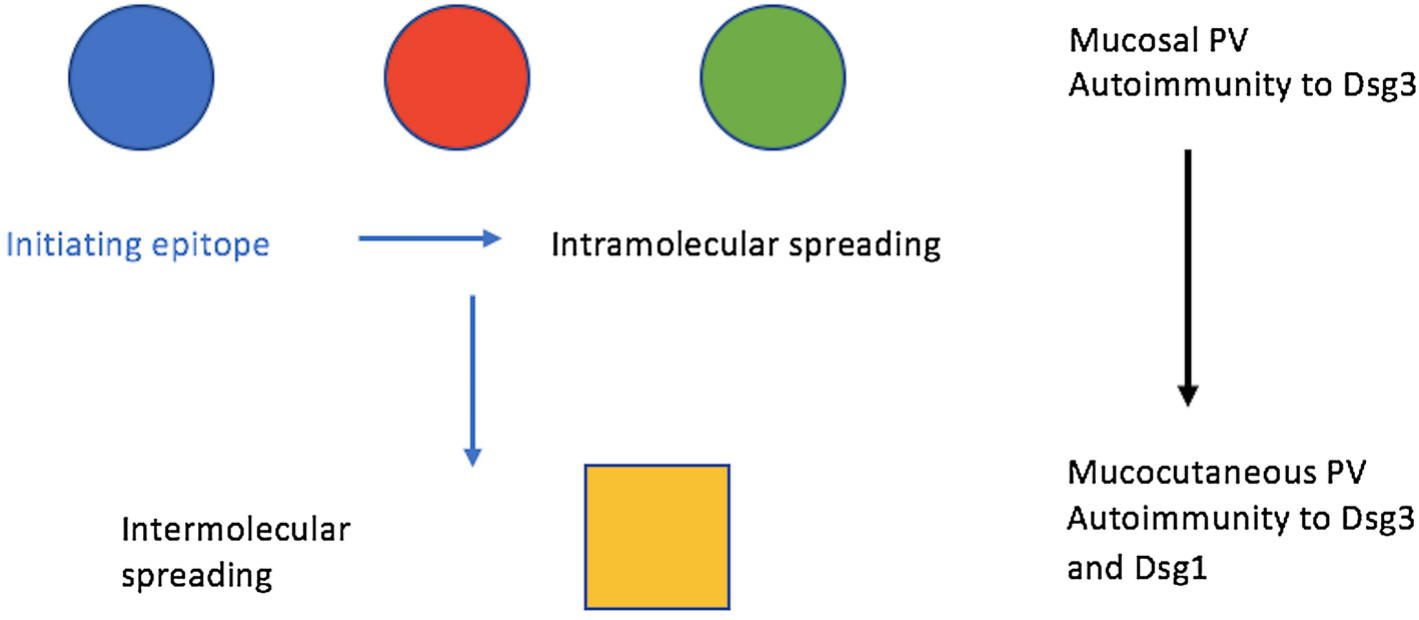
Epitope spreading in PV. The primary immune response from the initiating Dsg epitope (blue circle) can cause different Dsg3 epitopes (red and green circles) within the same protein to be exposed; this is known as intramolecular epitope spreading. In PV, this precedes intermolecular spreading where Dsg1 epitopes in a different protein are exposed (yellow square) and hence cutaneous lesions occur. [5, 24]

Transitions between the two pemphigus subtypes are a known phenomenon and therefore, it could be possible that a transition from PF to PV has occurred in our patient [2, 10, 18–20]. The process of transition may be explained by qualitative changes in desmoglein autoantibody profile [2, 18]. Exactly how this occurs is not fully known, however one proposed mechanism is epitope spreading [2, 21]. An epitope is the part of an antigen molecule to which an antibody attaches itself. In epitope spreading, the primary autoimmune response leads to tissue damage which causes a new epitope to be revealed, hence provoking a secondary autoimmune response [21, 22]. Epitope spreading can occur both within the same protein (intramolecular) (Fig. 7) and between distinct proteins within the same tissue (intermolecular) [5, 21]. PV is an example of both intramolecular and intermolecular epitope spreading (Fig. 8); in the earlier stages of the disease, patients tend to only display mucosal lesions but often progress to mucocutaneous involvement. This suggests that intermolecular epitope spreading has occurred from Dsg3 to Dsg1 and, as described previously, when both Dsg1 and Dsg3 are present no compensation can occur which results in blister formation in both mucosa and skin. In the initial stages of PV, when only anti-Dsg3 autoantibodies are present, the presence of Dsg1 throughout the entire epidermis compensates for the loss of Dsg3 in the basal layers and the skin is spared. However, in the mucosa, Dsg1 is present in too low amounts to compensate, and mucosal blisters occur [5–8].

With regards to intramolecular epitope spreading in PV (Fig. 7), it is suggested that in the two disease stages (mucosal PV and mucocutaneous PV) the Dsg3 autoantibodies recognise different Dsg3 epitopes. Specifically, studies have shown that Dsg3 autoantibodies in mucosal PV do not bind to human skin in indirect immunofluorescence however in mucocutaneous PV they do [5, 25]. The secondary Dsg3 epitope is present in skin and shows homology with Dsg1 [5, 21]. It has been demonstrated that this Dsg3 intramolecular epitope spreading precedes the intermolecular epitope spreading from Dsg3 to Dsg1 [5] (Fig. 8). In the case of PF transforming to PV, it may be that autoantibodies to Dsg1 only are originally present, but the tissue damage to the skin causes ‘hidden’ Dsg3 to be ‘revealed’ to the immune system, provoking the production of autoantibodies to Dsg3 [10, 21, 26]. Epitope spreading occurs in several other autoimmune skin diseases, including epidermolysis bullosa acquisita, bullous pemphigoid, lichen planus pemphigoides and systemic lupus erythematosus [21].

Transitions between PF and PV are rare; a literature search using *Pubmed, Ovid* and *Web of Science* using the search terms ‘pemphigus foliaceus’ and ‘pemphigus vulgaris’ and ‘transition’ or ‘shift’ revealed a total of 19 relevant papers describing 24 cases of PV to PF transition, and only 6 cases of PF to PV transition. One case transitioned from PV to PF and then back to PV [2]. Of these 19 papers, 16 (equaling 26 transition cases) used either immunoblotting, enzyme-linked immunosorbent assay (ELISA) or both, to detect autoantibodies and confirm shifts between Dsg1 and Dsg3 [2, 10, 13, 20, 27–30]. Both techniques are used to identify target proteins, however, studies have shown ELISA to be more highly specific and sensitive for detecting autoantibodies in the sera of patients with PF and PV [2, 31–34]. PF sera only reacts with Dsg1 whereas PV sera can react with both Dsg1 and Dsg3 [2]. Unfortunately ELISA or immunoblotting was not used in our patient; therefore, it is difficult to confirm if a true transition from PF to PV has occurred. However, as PF only targets Dsg1, and subsequently the patient developed lesions in the oral mucosa (which predominantly contains Dsg3) [4, 9, 10], there is evidence of a shift in autoantibody profile. It may be the case that if a repeat biopsy from the scalp was performed, it would reveal PV; however, there is no clinical justification to do so currently as it would not change the patient management.

It is interesting that in addition to PF and oral PV the patient was also clinically diagnosed with genital lichen planus. Unfortunately, there is no histological confirmation of this diagnosis, as biopsy was not deemed to be warranted due to the characteristic clinical features of the glans penis and because the result would not alter our current management. Therefore, it could be that the genital lesions in fact represent genital PV, especially given that the oral lesions also have a clinically lichenoid appearance (Fig. 4). Furthermore, within the PV buccal mucosa biopsy there was a distinct area of hyperkeratosis with an associated band-like infiltrate resembling a lichenoid tissue reaction, however the significance of this is unknown. Interestingly, within the literature there are cases of oral lichen planus with circulating anti-Dsg3 antibodies, which are typically characteristic of PV [35, 36]. The pathogenic role of these antibodies in lichen planus however, is not yet understood.

Regarding management, it was crucial that a multidisciplinary approach was taken; hence the patient is seen regularly on the joint Oral Medicine/Dermatology clinics. Treatment has been tailored to both the patient’s oral and cutaneous symptoms, with one of the main challenges being reaching a stable dose of MMF appropriate for all aspects of the patient’s condition. Over the past 9 years, the dose has varied from 500 mg twice a day to 1.5 g twice a day, with various combinations in between. With these regular dose alterations, it has been vital to ensure that appropriate blood monitoring has been adhered to prevent side effects such as neutropenia and leucopenia [37]. With the patient travelling a significant distance to our clinic, this has required close liaison and a shared care protocol with the General Medical Practitioner.

## Conclusion

This case report describes an interesting and rare case of concomitant PF and oral PV. It is possible that this represents an uncommon case of transition from PF to PV, however without confirmation with ELISA or immunoblotting, a true transition is difficult to confirm. The process of transition may be explained by the mechanism of epitope spreading, which occurs in other autoimmune skin diseases. It is essential that patients with mucocutaneous conditions are managed with a multidisciplinary approach and tailored therapy.
